# Positive lymph node retrieval ratio optimises patient staging in colorectal cancer

**DOI:** 10.1038/sj.bjc.6605049

**Published:** 2009-04-28

**Authors:** S J Moug, J D Saldanha, J R McGregor, M Balsitis, R H Diament

**Affiliations:** 1 Department of General Surgery, Crosshouse Hospital, Kilmarnock Road, Kilmarnock, Scotland, KA2 0BE, UK; 2 Department of Pathology, Crosshouse Hospital, Kilmarnock Road, Kilmarnock, Scotland, KA2 0BE, UK

**Keywords:** staging, colorectal cancer, positive lymph node ratio

## Abstract

Alternative lymph node (LN) parameters have been proposed to improve staging in colorectal cancer. This study compared these alternative parameters with conventional TNM staging in predicting long-term survival in patients undergoing curative resection. A total of 295 consecutive patients (mean age 70 years; range 39–95; s.d. 10.4) underwent resection for colorectal cancer from 2001 to 2004. Age, sex, primary tumour site, TNM stage and chemotherapy/radiotherapy were recorded. Patients with colon and rectal cancers were analysed separately for LN parameters: LN total; adequate LN retrieval (⩾12) and inadequate (<12); total number of negative LN; total number of positive LN and the ratio of positive LN to total LN (pLNR). Univariate and multivariate survival analysis was performed. The median number of LN retrieved was 10 (1–57) with adequate LN retrieval in 147 cases (49.8%). For each T and N stage, inadequate LN retrieval did not adversely affect long-term survival (*P*>0.05). On multivariate analysis, only pLNR was an independent predictor of overall survival in both colon and rectal cancers (HR 11.65, 95% CI 5.00–27.15, *P*<0.001 and HR 13.40, 95% CI 3.64–49.10, *P*<0.001, respectively). Application of pLNR subdivided patients into four prognostic groups. Application of the pLNR improved patient stratification in colorectal cancer and should be considered in future staging systems.

Colorectal cancer is one of the commonest malignancies in the United Kingdom, with the presence of lymph node metastases being one of the most important prognostic determinants. Currently, patients with colorectal cancer are staged used the TNM staging system that divides patients into prognostic groups according to the growth of the primary tumour, presence of lymph node metastases and evidence of distant metastatic spread. Lymph node status is determined by the number of positive lymph nodes retrieved with prognosis declining as the number of positive lymph nodes increases: node negative (N0); node positive with 1–3 nodes positive (N1) and node positive with ⩾4 nodes positive (N2; Sobin and Wittekind, 2002). However, some researchers believe that TNM may not result in optimal staging and have proposed alternative lymph node parameters.

One of these alternative parameters focuses on the overall number of lymph nodes retrieved. Initial work was performed on node-negative disease, where researchers found that as the number of lymph nodes examined increased, the disease-free and overall survival improved (Caplin *et al*, 1998; Prandi *et al*, 2002; Le Voyer *et al*, 2003; Swanson *et al*, 2003; Berger *et al*, 2005; Edler *et al*, 2007; Wong *et al*, 2007). Attention turned to node-positive disease with a recent systematic review by the National Cancer Institute concluding after analysing over 60 000 patients that there was a positive association between increasing number of lymph nodes retrieved and survival in stage II and stage III colon cancer (Chang *et al*, 2007). As a result, many researchers have tried to determine the minimum number of lymph nodes that should be retrieved to ensure accurate staging in colonic cancer, but to date no consensus has been achieved with recommendations varying from as few as 6 to as many as 40 (Hernanz *et al*, 1994). Currently, the National Cancer Institute and the Royal College of Pathologists recommend a minimum retrieval of 12 (Nelson *et al*, 2001; Royal College of Pathologists, 2007).

In direct comparison to TNM staging, Johnson *et al* (2006), analysed the relationship between the number of negative lymph nodes retrieved and long-term survival. Over 20 000 patients with stage III colon cancer who had undergone curative resection were assessed with stage IIIB and IIIC patients having improved long-term survival as the number of negative nodes increased. Unfortunately, this phenomenon was not replicated in stage IIIA patients, leading the authors to state further research is required to clarify the relationship between total number of negative lymph nodes and survival.

The ratio between metastatic and examined lymph nodes (the positive lymph node ratio; pLNR) has been shown to identify prognostic subgroups within gastric and oesophageal cancer patients (Marchet *et al*, 2007; Mariette *et al*, 2008). In one of these studies, the authors retrospectively analysed approximately 2000 patients that had undergone radical resection for gastric carcinoma at different centres. After multivariate analysis pLNR, but not N stage, was an independent prognostic factor that was also independent of the extent of lymphadenectomy. Only a few studies have analysed pLNR in colon cancer, with prognosis declining as pLNR increased (De Ridder *et al*, 2006; Lee *et al*, 2007). However, in one of these studies, pLNR was only found to be significant if the lymph node retrieval was >15, suggesting adequate lymph node retrieval to be of greater importance than pLNR.

Unlike patients with colonic cancer, patients with rectal cancers are routinely considered for neoadjuvant chemo/radiotherapy before undergoing surgical resection. In addition to shrinking the primary tumour and reducing the risk of local recurrence after surgery, radiotherapy may also decrease the number of local lymph nodes (Wichmann *et al*, 2002). As a consequence there has been limited work assessing the minimum lymph node retrieval number in rectal cancer, with no definitive figure proposed. Despite this, the Royal College of Pathologists guidelines state that a mean number of 12 lymph nodes should be retrieved with no specific guidelines for rectal or neoadjuvant therapy and the College of American Pathologists also suggest a minimum retrieval of 12 lymph nodes (Royal College of Pathologists, 2007; National Comprehensive Cancer Network, 2009).

This study aimed to compare these alternative approaches to staging of lymph nodes in colorectal cancer and to clarify their influence on long-term survival in node-negative and node-positive patients undergoing curative resection.

## Materials and Methods

A total of 295 consecutive patients that underwent curative resection for colorectal cancer in two centres between 2001 and 2004 were identified from a departmental prospective database. Curative resection was defined by pathological clear margins on the resected specimen with no evidence of metastatic spread on preoperative imaging of chest, abdomen and pelvis. After resection, the patients entered a follow-up surveillance programme with out-patient clinic appointments: 3 and 6 months postoperative; then yearly thereafter. At each appointment, they underwent clinical history and examination with testing for carcinoembryonic antigen, liver function and bone markers. In addition, they had a surveillance colonoscopy and contrast enhanced CT at 1 and 5 years. At the time of this study, all patients were participating in the follow-up programme with a median follow-up of 4 years (range 0.1–6.9 years).

Each patient had their age, sex, site of primary tumour (colon or rectum) and their TNM stage recorded. Neoadjuvant chemotherapy and/or radiotherapy and adjuvant chemotherapy were documented. In relation to lymph node retrieval, the following variables were calculated separately for colonic and rectal surgery: the total number of lymph nodes (LN total) and subsequently, adequate retrieval (⩾12) and inadequate (<12); the total number of negative lymph nodes (LN negative) and total number of positive lymph nodes (LN positive) and the ratio of positive lymph nodes to total lymph nodes (pLNR).

To determine the influence of lymph node retrieval on long-term outcome, univariate and multivariate survival analysis was performed using a stepwise backward procedure to derive a final model of the variables that had a significant independent relationship with survival. To remove a variable from the model, the corresponding *P* value had to be greater than 0.10. Analysis was performed using SPSS software version 15.0 (SPSS Inc., Chicago, IL, USA)

## Results

### Patient demographics and TNM staging

The mean age in this study was 70 years (range 39–95, s.d. 10.4) with a slight male preponderance (163 men, 132 women). Colonic resections accounted for the majority of resections (*n*=195, 66.1%) with 84 patients (28.5%) having undergone any form of chemo/radiotherapy.

Tables 1 and 2 display the demographics, TNM staging and the number of patients undergoing chemo/radiotherapy for colon and rectal cancers.

### Lymph node retrieval

Of the 295 cases, adequate lymph node retrieval was only achieved in 147 cases (49.8%). For colon cancers, adequate retrieval occurred in 48.2% of patients whereas in rectal, 53% of patients had at least 12 lymph nodes removed. For each T and N stage, inadequate lymph node retrieval did not adversely affect long-term survival (Kaplan–Meier log-rank test *P*>0.050).

In relation to the other lymph node parameters, total number of lymph nodes retrieved and total number of negative lymph nodes did not influence overall survival in either colon or rectal cancers, whereas the remaining lymph node variables analysed, including N stage, achieved statistical significance (Table 3). On multivariate analysis, only pLNR was an independent predictor of overall survival in both colon and rectal cancers (HR 11.65, 95% CI 5.00–27.15, *P*<0.001 and HR 13.40, 95% CI 3.64–49.10, *P*<0.001, respectively).

Inclusion of age into the multivariate model for colon cancers found both pLNR and age to be independent predictors of survival (HR 11.89, 95% CI 5.13–27.53, *P*<0.001 and HR 1.88, 95% CI 1.17–3.03, *P*=0.008, respectively).

### Subdivision of patients according to pLNR

**Figure 1:**
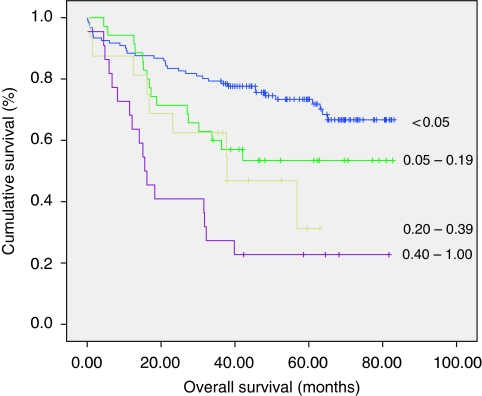
Differences in overall survival in patients undergoing curative resection for colon cancer when classified by positive lymph node ratios.

**Figure 2:**
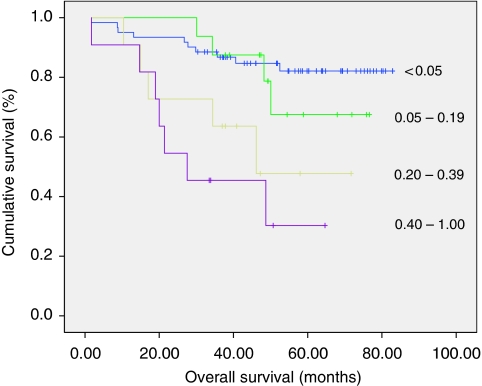
Differences in overall survival in patients undergoing curative resection for rectal cancer when classified by positive lymph node ratios.

The pLNR was subdivided into four groups according to a previous work (Berger *et al*, 2005): <0.05; 0.05–0.19; 0.20–0.39 and 0.40–1.0. In both colon and rectal cancer patients, overall survival significantly decreased as pLNR increased (*P*<0.001; Figures 1 and 2).

## Discussion

Determination of the optimal approach to quantifying lymph node status in colorectal cancer will ensure accurate patient staging, allowing appropriate adjuvant treatment planning and calculation of long-term prognosis. This study is one of the first to directly compare several of the proposed approaches in colon and rectal cancer, finding the positive lymph node ratio to be the optimal staging modality.

The total number of lymph nodes retrieved, total number of negative nodes and total number of positive nodes have been proposed by different researchers to optimally stage patients with colon cancer. In this study we analysed rectal cancers as well as colon cancers and found that none of these factors were significant on multivariate analysis. In addition, inadequate lymph node retrieval, as defined by NCI, occurred in approximately 50% of cases in this study. Despite this, no detriment to long-term survival was found in either node-negative or node-positive patients.

There are many factors potentially influencing lymph node retrieval including grade and specialty of surgeon, neoadjuvant radiotherapy and/ or chemotherapy and technique of analysis by the pathologist. There are also naturally occurring variables, specifically the age and sex of the patient. Lastly, the type of resection appears to be important with right-sided resections potentially yielding higher numbers of lymph nodes. With all these variables it is not surprising that the above lymph node parameters have not been found to optimally stage colorectal cancer patients. Indeed, this study is not the only one to document no detriment to survival with inadequate node retrieval. A recent study in 2007 looked at over 30 000 patients from the National Surveillance, Epidemiology and End-Results (SEER) Medicare-linked database. These patients were from four hospitals that had different lymph node retrieval rates, and despite the disparity, the authors found no significant survival difference (Wong *et al*, 2007).

The positive lymph node ratio may overcome some of the limitations of the other parameters as it divides node-positive patients into ranges, although not relying on one outcome variable. This may also overcome the difficulty of proposing a minimum lymph node retrieval number in rectal cancer patients that have undergone neoadjuvant therapy. In addition, it changes prognostic stratification. In patients with colon cancer in this study that were staged as N1, the 5-year survival was 47.2%. Application of the positive lymph node ratio to this group subdivides these patients: in those patients with the lowest positive lymph node ratios, their 5-year survival has improved to 58%, and in those with the highest positive lymph node ratio, their survival has reduced to 22%. For N2 patients, the 5-year survival difference between the lowest and highest lymph node ratios was 33%. This prognostic stratification change has also been commented on by the two previous papers assessing positive lymph node ratio in colon cancer.

Berger *et al* (2005) assessed this ratio in patients with stage II and stage III colonic cancer that had been specifically recruited for a chemotherapy trial. The authors found an increasing positive lymph node ratio resulted in poorer overall and disease-free survival. Furthermore, 5-year survival difference of 19% in N1 patients between the lowest and the highest ratios was documented that increased to 28% in N2 patients. However, the influence of positive lymph node ratio was not significant when lymph node retrieval was <15, leading the authors to conclude that lymph node retrieval is the more important factor. Our study methodology differs, in that we have analysed for any effect of a patient receiving chemotherapy, included rectal cancers and had a high rate of inadequate lymph node retrieval. However, we still found positive lymph node ratio to be independently prognostic in all patients, something that N staging could not achieve on multivariate analysis.

Our findings are supported by De Ridder *et al* (2006), who analysed patients with node-positive colonic and rectal cancers. This time, only two subdivisions were made with a pLNR of 0.4 taken as the threshold: patients with ratios greater than 0.4 had a 5-year survival of 25% *vs* 56% in the groups with lower ratios. This was compared to the N1 and N2 staged groups that had 5-year survival of 54 and 28%, respectively. These results led the authors to conclude that application of the positive lymph node ratio led to improved prognostic separation and should be used in future staging systems. As the authors only assessed two subdivisions, no comment can be made on whether greater disparity would have been found with further subdivisions.

Further work is underway to determine the appropriate subdivisions of the positive lymph node ratio in node-positive patients. It may be that the four subdivisions used here can be optimised, providing more prognostic information for clinicians and patients. Also, the application of the ratio in node-negative patients requires clarification. Perhaps it may have a role in identifying ‘high risk’ patients that should be considered for chemotherapy.

## Conclusion

Calculation of the positive lymph node ratio optimises patient staging and alters patient stratification, irrespective of lymph node retrieval numbers in both colon and rectal cancers.

## Figures and Tables

**Table 1:** Patient demographics, TNM staging, chemotherapy and their influence on long-term survival in patients undergoing curative resection for colon cancer

	***n***		**5-year survival (%)**	***P* value**
*Age*				
39–70 years	84	43.1	71.5	0.013^a^
70–92 years	111	56.9	55.2	
				
*Sex*				
Male	100	51.3	66.3	0.090
Female	195	48.7	60.7	
				
*T stage*				
T1	15	7.7	80.0	<0.001^a^
T2	20	10.3	71.0	
T3	105	53.8	65.1	
T4	55	28.2	35.0	
				
*N stage*				
N0	120	61.5	72.4	<0.001^a^
N1	49	25.1	47.2	
N2	26	13.3	22.9	
				
*Chemotherapy*				
Yes	53	26.7	58.7	0.459
No	143	73.3	65.7	

a Indicates log-rank test.

**Table 2:** Patient demographics, TNM staging, chemo/radiotherapy and their influence on long-term survival in patients undergoing curative resection for rectal cancer

	***n***	****	**5-year survival (%)**	***P* value**
*Age*				
39–70 years	57	57.0	76.0	0.151
70–92 years	43	43.0	63.4	
				
*Sex*				
Male	63	63.0	66.2	0.246
Female	37	37.0	77.8	
				
*T stage*				
T1	10	10.0	90.0	0.050
T2	29	29.0	86.7	
T3	53	53.0	58.0	
T4	8	8.0	75.0	
				
*N Stage*				
N0	60	60.0	81.6	0.040^a^
N1	22	22.0	60.6	
N2	18	18.0	43.5	
				
*Chemotherapy/radiotherapy*				
Yes^b^	31	31.0	67.0	0.537
No	69	69.0	72.0	

a Indicates log-rank test.

b Neoadjuvant therapy was performed in 21 cases.

**Table 3:** The influence of lymph node retrieval parameters on long-term survival after curative colonic and rectal cancer resections: univariate and multivariate analysis

	**Colonic resections**			**Rectal resections**		
	**Univariate *P* value**	**Multivariate *P* value**	**HR (95% CI)**	**Univariate *P* value**	**Multivariate *P* value**	**HR (95% CI)**
LN total	0.169			0.251		
Adequate LN retrieval	0.065			0.572		
LN negative	0.065			0.065		
LN positive	<0.001^*^	0.912	0.99 (0.87–1.13)	<0.001^*^	0.839	0.96 (0.65–1.43)
Positive LNR	<0.001^*^	<0.001^*^	11.65 (5.00–27.15)	<0.001^*^	<0.001^*^	13.4 (3.64–49.10)
N stage	<0.001^*^	0.642	1.13 (0.67–1.89)	0.004^*^	0.640	1.2 (0.54–2.72)

* *P*<0.05

## References

[bib1] Berger AC, Sigurdson ER, LeVoyer T, Hanlon A, Mayer RJ, Macdonald JS, Catalano PJ, Haller DG (2005) Colon cancer survival is associated with decreasing ratio of metastatic to examined lymph nodes. J Clin Oncol 23: 8706–8712 16314630 10.1200/JCO.2005.02.8852

[bib2] Caplin S, Cerottini JP, Bosman FT, Constanda MT, Givel JC (1998) For patients with Dukes’ B (TNM Stage II) colorectal carcinoma, examination of six or fewer lymph nodes is related to poor prognosis. Cancer 83: 666–672 9708929

[bib3] Chang GJ, Rodriguez-Bigas MA, Skibber JM, Moyer VA (2007) Lymph node evaluation and survival after curative resection of colon cancer: systematic review. J Natl Cancer Inst 99: 433–441 17374833 10.1093/jnci/djk092

[bib4] De Ridder M, Vinh-Hung V, Van Nieuwenhove Y, Hoorens A, Sermeus A, Strome G (2006) Prognostic value of the lymph node ratio in node positive colon cancer. Gut 55: 1681 10.1136/gut.2006.104117 PMC1860091 17047131

[bib5] Edler D, Ohrling K, Hallstrom M, Karlberg M, Ragnhammar P (2007) The number of analyzed lymph nodes—a prognostic factor in colorectal cancer. Acta Oncol 46: 975–981 17917828 10.1080/02841860701203537

[bib6] Hernanz F, Revuelta S, Redondo C, Madrazo C, Castillo J, Gomez-Fleitas M (1994) Colorectal adenocarcinoma: quality of the assessment of lymph node metastases. Dis Colon Rectum 37: 373–376 8168417 10.1007/BF02053600

[bib7] Johnson PM, Porter GA, Ricciardi R, Baxter NN (2006) Increasing negative lymph node count is independently associated with improved long-term survival in stage IIIB and IIIC colon cancer. J Clin Oncol 24: 3570–3575 16877723 10.1200/JCO.2006.06.8866

[bib8] Le Voyer TE, Sigurdson ER, Hanlon AL, Mayer RJ, MacDonald JS, Catalano PJ, Haller DG (2003) Colon cancer survival is associated with increasing number of lymph nodes analyzed: a secondary analysis of intergroup trial INT-0089. J Clin Oncol 21: 2912–2919 12885809 10.1200/JCO.2003.05.062

[bib9] Lee HY, Choi HJ, Park KJ, Shin JS, Kwon HC, Roh MS, Kim C (2007) Prognostic significance of metastatic lymph node ratio in node-positive colon carcinoma. Ann Surg Oncol 14: 1712–1717 17253102 10.1245/s10434-006-9322-3

[bib10] Marchet A, Mocellin S, Ambrosi A, Morgagni P, Garcea D, Marrelli D, Roviello F, de Manzoni G, Minicozzi A, Natalini G, De Santis F, Baiocchi L, Coniglio A, Nitti D (2007) The ratio between metastatic and examined lymph nodes (N Ratio) is an independent prognostic factor in gastric cancer regardless of the type of lymphadenectomy: results of an Italian multicentric study in 1853 patients. Ann Surg 245: 543–552 17414602 10.1097/01.sla.0000250423.43436.e1 PMC1877031

[bib11] Mariette C, Piessen G, Briez N, Triboulet JP (2008) The number of metastatic lymph nodes and the ratio between metastatic and examined lymph nodes are independent prognostic factors in oesophageal cancer regardless of neoadjuvant chemoradiation or lympadenectomy extent. Ann Surg 247: 365–371 18216546 10.1097/SLA.0b013e31815aaadf

[bib12] National Comprehensive Cancer Network (2009) National Comprehensive Cancer Network Clinical Practice Guidelines in Oncology. Rectal Cancer, http://misc.medscape.com/images/586/417/rectal.pdf 10.6004/jnccn.2009.0057 19755047

[bib13] Nelson H, Petrelli N, Carlin A, Couture J, Fleshman J, Guillem J, Miedema B, Sargent D (2001) National Cancer Institute Expert Panel. Guidelines 2000 for colon and rectal cancer surgery. J Natl Cancer Inst 93: 583–596 11309435 10.1093/jnci/93.8.583

[bib14] Prandi M, Lionetto R, Bini A, Francioni G, Accarpio G, Anfossi A, Ballario E, Becchi G, Bonilauri S, Carobbi A, Cavaliere P, Garcea D, Giuliani L, Morziani E, Mosca F, Mussa A, Pasqualini M, Poddie D, Tonetti F, Zardo L, Rosso R (2002) Prognostic evaluation of stage B colon cancer patients is improved by an adequate lymphadenectomy: results of a secondary analysis of a large scale adjuvant trial. Ann Surg 235: 458–463 11923600 10.1097/00000658-200204000-00002 PMC1422459

[bib15] Royal College of Pathologists (2007) Standards and Dataset for Reporting Cancers: Dataset for colorectal cancer (2nd edition). http://www.rcpath.org/resources/pdf/G049-ColorectalDataset-Sep07.pdf

[bib16] Sobin LH, Wittekind CH (2002) TNM Classification of Malignant Tumours. New York: Wiley-Liss

[bib17] Swanson RS, Compton CC, Stewart AK, Bland KI (2003) The prognosis of T3N0 colon cancer is dependent on the number of lymph nodes examined. Ann Surg Oncol 10: 65–71 12513963 10.1245/aso.2003.03.058

[bib18] Wichmann MW, Muller C, Meyer G, Strauss T, Hornung HM, Lau-Werner U, Angele MK, Schildberg FW (2002) Effect of preoperative radiochemotherapy on lymph node retrieval after resection of rectal cancer. Arch Surg 137: 206–210 11822961 10.1001/archsurg.137.2.206

[bib19] Wong SL, Hollenback BK, Morris AM, Baser O, Birkmeyer JD (2007) Hospital lymph node examination rates and survival after resection for colon cancer. J Am Med Assoc 298: 2149–2154 10.1001/jama.298.18.2149 18000198

